# rTg4510 Tauopathy Mice Exhibit Non-Spatial Memory Deficits Prevented by Doxycycline Treatment

**DOI:** 10.3390/brainsci15111183

**Published:** 2025-10-31

**Authors:** Yasushi Kishimoto, Takashi Kubota, Kentaro Nakashima, Yutaka Kirino

**Affiliations:** 1 Laboratory of Physical Chemistry, Faculty of Pharmaceutical Sciences, Teikyo University, Tokyo 173-8605, Japan; 2 Laboratory of Neurobiophysics, Kagawa School of Pharmaceutical Sciences, Tokushima Bunri University, Sanuki 769-2193, Japan; t.kubota@thu.ac.jp (T.K.); nkentaro@kph.bunri-u.ac.jp (K.N.); 3 Institute of Neuroscience, Tokushima Bunri University, Sanuki 769-2193, Japan; 4 Laboratory of Basic Pharmaceutical Education, Faculty of Pharmaceutical Sciences, Teikyo Heisei University, Tokyo 164-8530, Japan

**Keywords:** Alzheimer’s disease, contextual fear conditioning, doxycycline, tauopathy, trace eyeblink conditioning, non-spatial memory, rTg4510 mouse

## Abstract

**Background:** Hyperphosphorylated tau accumulation and neurofibrillary tangles (NFTs) are hallmarks of tauopathies, including Alzheimer’s disease (AD), and are strongly associated with cognitive decline. The rTg4510 mouse model, which expresses mutant human tau (P301L), develops progressive tauopathy in the absence of amyloid-β pathology, providing a valuable tool for investigating tau-driven neurodegeneration. Previous studies have demonstrated spatial and object-recognition memory deficits at six months of age, which can be prevented by doxycycline (DOX)-mediated suppression of tau expression. However, it remained unclear whether non-spatial hippocampal learning, particularly temporal associative learning, would be similarly affected. **Methods:** We evaluated six-month-old rTg4510 mice with or without DOX treatment. To control for potential motor confounds, we first assessed spontaneous home cage activity. We then tested hippocampus-dependent non-spatial learning using two paradigms: trace eyeblink conditioning (500-ms trace interval) and contextual fear conditioning. **Results:** General motor function remained intact; however, rTg4510 mice without DOX treatment exhibited increased rearing behavior. These mice demonstrated significant deficits in trace eyeblink conditioning acquisition, with particularly clear impairment on the final day of training. Contextual fear conditioning showed milder deficits. Analysis of response peak latency revealed subtle temporal processing abnormalities during early learning. Two months of DOX treatment initiated at four months of age prevented these learning deficits, confirming their association with tau overexpression. **Conclusions:** Our findings demonstrate that rTg4510 mice exhibit deficits in non-spatial temporal associative learning alongside previously reported spatial and object-recognition impairments. Trace eyeblink conditioning serves as a sensitive behavioral assay for detecting tau-related hippocampal dysfunction, and the prevention of learning deficits by DOX treatment highlights its potential utility as a translational biomarker for evaluating tau-targeted interventions.

## 1. Introduction

Tauopathies are a group of neurodegenerative disorders characterized by the abnormal aggregation of hyperphosphorylated tau protein into intracellular neurofibrillary tangles (NFTs) [1]. Among these disorders, Alzheimer’s disease (AD) is the most prevalent and clinically significant, featuring both tau pathology and extracellular amyloid-β (Aβ) plaques [2]. Although Aβ has long been considered the primary driver of AD pathogenesis, supported by genetic and biochemical evidence from amyloid precursor protein (APP) and presenilin mutations, accumulating research indicates that tau pathology is a critical determinant of synaptic dysfunction and cognitive decline [3].

Recent clinical trials of anti-Aβ immunotherapies such as lecanemab have shown substantial plaque reduction but only modest cognitive benefit [4,5], consistent with clinicopathological findings that amyloid burden correlates weakly with cognitive impairment [6]. These results underscore the need to focus on tau-related mechanisms of neurodegeneration [7].

The rTg4510 mouse model expresses mutant human tau (P301L) and develops progressive tauopathy without Aβ pathology, reproducing key aspects of tau-driven neurodegeneration and memory deficits [8,9]. Earlier studies demonstrated that suppression of tau expression improves memory performance and reduces pathology, which otherwise progresses with age [10,11].

We recently reported that spatial and object-recognition memory impairments emerge by six months of age but can be prevented by doxycycline (DOX)-mediated suppression of tau expression [12], highlighting tau’s central role in learning and memory dysfunction. However, hippocampus-dependent memory extends beyond spatial domains. Classic studies have established the hippocampus as crucial for multiple forms of declarative memory [13], involving distinct but partially overlapping neural circuits [14].

For instance, the dorsal hippocampus and medial entorhinal cortex primarily support spatial memory, whereas the ventral hippocampus and lateral entorhinal cortex preferentially mediate non-spatial and emotional tasks [15,16,17]. Moreover, hippocampal CA1 and CA3 neurons encode both spatial and temporal information that support cross-modal and temporally precise associative learning [18,19,20,21]. Thus, while both spatial and non-spatial paradigms engage the hippocampus, they depend on distinct networks and synaptic mechanisms.

Given the recent advances in tau-targeted therapeutics, it is essential to develop behavioral assays that sensitively detect tau-related dysfunction. Unlike Aβ, tau pathology propagates trans-synaptically via extracellular tau species, spreading across brain networks and accelerating disease progression [22,23]. Fundamental studies of tau aggregation and propagation [24] have guided the development of immunotherapies designed to intercept tau spread, which show strong preclinical promise [25] but mixed clinical outcomes [26].

Such developments highlight the need for translational behavioral paradigms that specifically capture tau-dependent deficits. To date, it remains unclear whether tau pathology disrupts non-spatial hippocampal learning in parallel with spatial memory deficits. In this study, we examined whether tau pathology impairs non-spatial hippocampus-dependent memory alongside previously reported spatial deficits in rTg4510 mice. By directly comparing long-trace eyeblink conditioning and contextual fear conditioning, we evaluated the sensitivity of these paradigms to tau-related dysfunction. Our findings demonstrate that long-trace eyeblink conditioning, in particular, provides a sensitive and reliable readout of tau-dependent impairments, supporting its potential as a translational biomarker of early cognitive decline in tauopathy models.

## 2. Materials and Methods

### 2.1. Animals

We generated Tg(Camk2a-tTA)1Mmay Fgf14^Tg(tetO-MAPT*P301L)4510Kha^/J (rTg4510) mice by crossing two Jackson Laboratory (Bar Harbor, ME, USA) lines: (1) Tg(Camk2a-tTA) mice (129S6.Cg-Tg(Camk2a-tTA)1Mmay/J), which express the tetracycline-controlled transactivator protein (tTA) under the CaMKIIα promoter, and (2) tet-responsive Tg mice (FVB-Fgf14-Tg(tetO-MAPT*P301L)4510Kha/J) carrying the human mutant P301L 4R0N tau isoform downstream of a tetracycline-responsive element. Six-month-old male and female rTg4510 mice and age-matched nontransgenic wild-type (WT) controls from the same breeding colony were used [12]. The male–female ratio was balanced across experimental groups to avoid sex bias. A total of 36 mice were included: 18 WT and 18 rTg4510. Each genotype was randomly assigned to either DOX-treated or untreated groups (*n* = 9 per group), yielding four experimental groups. Sample sizes followed our laboratory’s established protocols for these behavioral assays and previous publications demonstrating reliable detection of cognitive deficits in transgenic mouse models.

Mice were housed four per cage in a temperature- and light-controlled environment (12-h light/dark cycle, lights on at 8:00 a.m.) with ad libitum access to food and water. All procedures complied with the guidelines of the Institutional Animal Investigation Committee at Tokushima Bunri University and the United States National Institutes of Health Guide for the Care and Use of Laboratory Animals. DOX was mixed into standard rodent chow (D13110902, Research Diets, New Brunswick, NJ, USA) and provided ad libitum beginning at 4 months of age. DOX administration continued throughout the two-month period preceding behavioral testing at 6 months. This regimen was identical to that used in our previous study [12], where molecular efficacy was documented.

### 2.2. Behavioral Tests

All mice underwent the same sequence of behavioral assessments: spontaneous locomotor activity assessment, followed by trace eyeblink conditioning, and contextual fear conditioning. Each task was separated by an interval of approximately 5 days to allow recovery and minimize carryover effects. All behavioral assessments were conducted during the 6th month of age.

#### 2.2.1. Spontaneous Physical Activities

To assess general motor function and exclude locomotor impairments as a confound, spontaneous home-cage activity was evaluated as previously described [27,28]. Mice were individually placed in clean home cages (21 × 31 × 12 cm) and video-recorded for 1 h during the light phase (between 10:00 and 15:00). Video data were analyzed using the HomeCageScan system (CleverSys, Inc., Reston, VA, USA) to quantify locomotor parameters, including rearing frequency and total distance traveled.

#### 2.2.2. Trace Eyeblink Conditioning

Long-trace eyeblink conditioning was performed following previously established methods [28,29,30], including surgical procedures, conditioning protocols, and criteria for conditioned response (CR) evaluation. Mice were anesthetized with ketamine (80 mg/kg, i.p.; Daiichi Sankyo, Tokyo, Japan) and xylazine (20 mg/kg, i.p.; Bayer Healthcare Pharmaceuticals, Berlin, Germany). Four Teflon-coated stainless-steel wires (100 μm diameter; A-M Systems, Sequim, WA, USA) were subcutaneously implanted into the left eyelid. Two wires delivered the unconditioned stimulus (US), and two recorded the electromyogram (EMG) from the orbicularis oculi (OO) muscle. A 352-ms tone (1 kHz, 80 dB) served as the conditioned stimulus (CS), and a 100-ms electrical shock (0.2 mA, 100 Hz, square pulses) served as the US. In the long-trace eyeblink conditioning paradigm, the CS and US were separated by a 500-ms trace interval (TI). Each session consisted of 90 CS-US paired trials and 10 CS-only trials (every tenth trial). Mice underwent 10 daily sessions. All experiments were performed by operators blinded to genotype. Response peak latency was defined as the time from CS onset to the maximum EMG amplitude occurring within the interval between CS onset and US onset, as previously described in detail [28,29,30]. Peak latency was analyzed on Days 4 and 10 to assess temporal characteristics of learned responses during early acquisition and final performance phases.

#### 2.2.3. Contextual Fear Conditioning

Conditioning was performed in operant chambers (26 cm length × 32 cm width × 21 cm height; CleverSys Operant Chamber) in sound-attenuating boxes (43 cm length × 46 cm width × 43 cm height). The chamber was equipped with a light and a speaker, and a floor composed of stainless-steel rods, through which a foot shock could be administered. All stimuli were controlled using FreezeScan TM1.0 system (Clever Sys, Inc., Reston, VA, USA). Conditioning was performed as previously described [31]. Mice were trained and tested on 2 consecutive days. In training on day 1, each mouse was placed in the operant chamber with constant house light illumination and allowed to explore for 3 min. After exploration, a 2 s foot shock [1.5 mA; US] was administered, and the mice were removed from the chamber 30 s later. 24 h after training, the mice were returned to the same chamber, and freezing behavior was recorded for 3 min (test trial). Freezing was defined as the absence of any movement except breathing. Freezing was measured using the FreezeScan video tracking system and software (CleverSys).

### 2.3. Statistical Analysis

Data were analyzed using Tukey–Kramer post hoc multiple comparisons following repeated-measures analysis of variance (ANOVA) in SPSS software 20 (IBM Corporation, Armonk, NY, USA). The threshold for statistical significance was set at *p* < 0.05. All data are presented as mean ± standard error of the mean (SEM).

### 2.4. Statement on the Use of Generative AI

In preparing the manuscript, generative artificial intelligence (Claude, OpenAI) was used to assist in improving the clarity of English phrasing, under the supervision of the authors.

## 3. Results

### 3.1. Spontaneous Physical Activity Assessment Reveals Preserved Motor Function with Increased Rearing Behavior in rTg4510 Mice Without DOX

**Figure 1:**
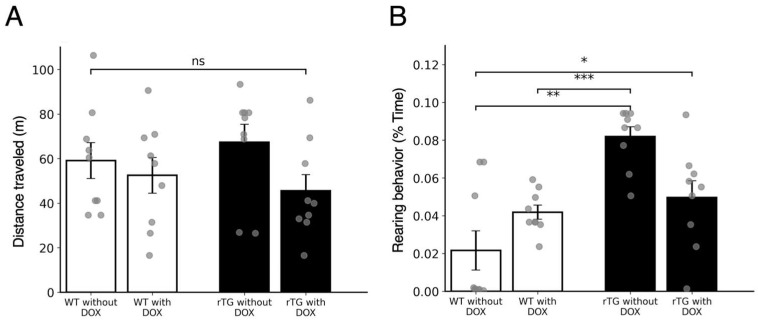
Spontaneous physical activities in the home cage. (**A**) Total distance traveled (m). rTg4510 mice without DOX showed the longest running distance among the groups, but no statistically significant differences were detected. (**B**) Rearing behavior (% time). One-way ANOVA revealed a significant group effect, and post-hoc Tukey’s test indicated that rTg4510 mice without DOX exhibited significantly increased rearing compared with WT without DOX, WT with DOX, and rTg4510 mice with DOX (*n* = 9 per group). * *p* < 0.05, ** *p* < 0.01, *** *p* < 0.001; “ns” indicates not significant. Bars represent mean ± SEM with individual data points overlaid.

To confirm that cognitive deficits were not secondary to motor impairment, we assessed spontaneous behavior in the home cage (Figure 1). Distance traveled tended to be higher in rTg4510 mice without DOX, although this did not reach statistical significance (Figure 1A; F(3, 32) = 1.42, *p* = 0.26; all post-hoc *p* > 0.2). On the other hand, rTg4510 mice without DOX treatment exhibited significantly increased rearing behavior compared with all other groups (Figure 1B; F(3, 31) = 9.53, *p* = 0.00013). Post-hoc analyses revealed that rTg4510 mice without DOX exhibited significantly increased rearing compared with WT without DOX (*p* < 0.001), WT with DOX (*p* < 0.01), and rTg4510 with DOX (*p* < 0.05). Other behavioral parameters (walking, jumping, hanging, and grooming) were unaffected. These results indicate that although rTg4510 mice display selective changes in exploratory behavior, overall motor function remains intact. Given this preservation of locomotor capacity despite these selective and mild behavioral changes, we proceeded to evaluate hippocampus-dependent learning paradigms in these mice.

### 3.2. rTg4510 Mice Show DOX-Dependent Deficits in Trace Eyeblink Conditioning

**Figure 2:**
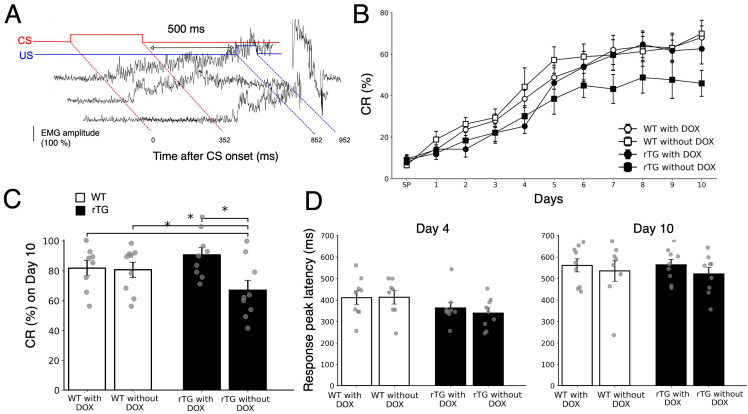
Trace eyeblink conditioning was impaired in rTg4510 mice without DOX. (**A**) Schematic representation of stimulus timing for the trace eyeblink conditioning paradigm used in the present study. A stimulus-free interval (500 ms) is present between the end of the conditioned stimulus (CS) and the onset of the unconditioned stimulus (US). Examples of three typical electromyographic (EMG) recordings from the orbicularis oculi (OO) muscle are shown below the CS–US timing diagram. (**B**) Acquisition curves of conditioned response (CR) across 10 daily sessions. Four groups of mice were subjected to trace eyeblink conditioning: WT with DOX (open circles; *n* = 9), WT without DOX (open squares; *n* = 8), rTg4510 with DOX (filled circles; *n* = 9), and rTg4510 without DOX (filled squares; *n* = 9). Data are presented as mean ± SEM. (**C**) Final learning performance on Day 10. Open bars and filled bars represent WT and rTg4510 mice, respectively; each genotype is shown with and without DOX treatment. Individual data points are superimposed on the bars. * *p* < 0.05 compared with all other groups (one-way ANOVA with post-hoc tests). (**D**) Response peak latency measured on Day 4 (**left panel**) and Day 10 (**right panel**). Open bars and filled bars represent WT and rTg4510 mice, respectively; each genotype is shown with and without DOX treatment. Individual data points are superimposed on the bars. Data are presented as the mean ± SEM.

To evaluate non-spatial hippocampal learning, we tested acquisition of trace eyeblink conditioning with a 500-ms trace interval in rTg4510 and WT mice with or without DOX treatment (Figure 2A). WT mice with or without DOX, as well as rTg4510 mice with DOX, successfully acquired CRs over 10 daily sessions, reaching approximately 60–70% CRs on day 10 (Figure 2B). In contrast, rTg4510 mice without DOX showed impaired learning, achieving less than 50% CRs even after 10 days of training.

Repeated-measures ANOVA across all sessions and all four groups showed a trend toward group differences (F(3, 31) = 2.59, *p* = 0.071), without a significant group × session interaction (F(27, 279) = 0.89, *p* = 0.63). To directly assess the effect of DOX treatment on rTg4510 mice, we performed a separate two-group repeated-measures ANOVA comparing rTg4510 with DOX versus rTg4510 without DOX, which revealed a significant main effect of group (F(1, 16) = 6.83, *p* = 0.019), with no significant interaction (F(9, 144) = 1.01, *p* = 0.43).

To further characterize final learning performance, we analyzed CR rates specifically on day 10 (Figure 2C). Post-hoc multiple comparisons revealed a statistically significant difference among the four groups (F(3, 31) = 4.53, *p* = 0.021), with rTg4510 mice without DOX showing significantly lower performance compared with all other groups. Thus, we confirmed that rTg4510 mice without DOX exhibited reduced CR rates compared with rTg4510 mice with DOX, indicating that the impaired temporal learning in rTg4510 mice was prevented by DOX treatment.

To examine the temporal characteristics of the learned responses, we analyzed response peak latency on Day 4 (early learning) and Day 10 (final performance) (Figure 2D). At the early stage of learning (Day 4), response peak latency was generally short across all groups, with both rTg4510 groups showing a trend toward earlier CR onset compared with WT mice; however, this difference did not reach statistical significance (one-way ANOVA, F(3, 31) = 1.70, *p* = 0.19). By the final day (Day 10), response peak latency had increased in all groups and converged to approximately 550 ms on average. A paired t-test across all subjects confirmed a significant overall increase in response peak latency from Day 4 to Day 10 (t_(27)_ = –8.24, *p* < 0.001), indicating that the initial trend for shorter latency in rTg4510 mice diminished as training progressed.

### 3.3. rTg4510 Mice Exhibit Mild DOX-Dependent Impairment in Contextual Fear Memory Retention

**Figure 3:**
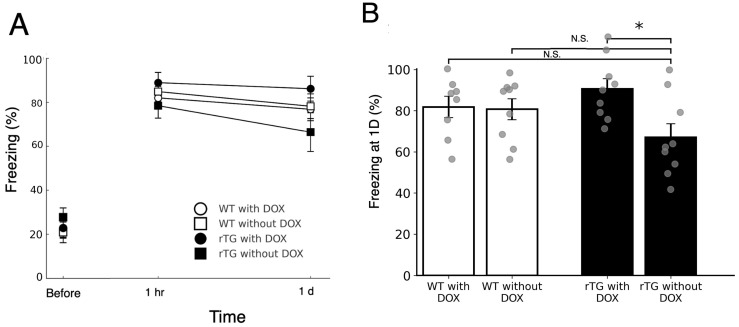
Contextual fear conditioning shows mild impairment in rTg4510 mice without DOX. (**A**) Freezing behavior before conditioning, 1 h after conditioning, and during the 24-h retention test in WT and rTg4510 mice with or without DOX (*n* = 9 per group). “Before” values are shown as isolated points, whereas 1 h and 1 d values are connected by lines. (**B**) Freezing levels at the 24-h retention test. ANOVA revealed a significant group effect, and post-hoc Tukey’s test indicated that rTg4510 mice without DOX froze significantly less than rTg4510 mice with DOX, while no significant differences were found compared with the WT groups. Bars represent the mean ± SEM with individual data points overlaid. * *p* < 0.05. N.S. indicate not significant.

We next examined contextual fear conditioning (Figure 3A). During habituation, rTg4510 mice without DOX did not differ from other groups in baseline exploration during the three-min pre-conditioning period (all *p* > 0.68). Repeated-measures ANOVA revealed a significant effect of genotype (F(3, 31) = 4.14, *p* = 0.014) without a significant genotype × time interaction effect (F(3, 31) = 0.45, *p* = 0.721; Figure 3A).

To further evaluate retention, freezing levels at 24 h were compared across groups (Figure 3B). Post hoc comparisons showed that rTg4510 mice without DOX froze significantly less than rTg4510 mice with DOX (*p* = 0.023), whereas no significant differences were observed relative to WT groups (*p* > 0.05). On the other hand, at the 1-h retention test, no group differences emerged (all *p* > 0.45). These results indicate that contextual fear memory retention was only mildly impaired in a DOX-dependent manner.

## 4. Discussion

The present study demonstrates that rTg4510 mice, a well-established tauopathy model, exhibit DOX-dependent deficits not only in spatial memory as previously reported [9], but also in hippocampus-dependent non-spatial tasks (Figure 2 and Figure 3). Specifically, trace eyeblink conditioning (Figure 2) revealed clear impairments at six months of age, whereas contextual fear conditioning (Figure 3) showed milder but detectable deficits. These findings extend prior demonstrating that tau pathology disrupts both spatial and non-spatial memory domains, supporting the view of widespread hippocampal dysfunction. Assessment of spontaneous physical activities (Figure 1) provided important validation for these cognitive findings. Although rTg4510 mice without DOX treatment exhibited increased rearing behavior, consistent with previous characterizations of this model [32], general motor function remained intact. Importantly, we did not observe anxiety-related behaviors such as grooming, which are commonly observed in anxiety models. This preservation of basic locomotor abilities, in the absence of overt anxiety-like behavior, confirms that the observed learning and memory deficits reflect specific cognitive dysfunction rather than secondary effects of motor impairment or emotional disturbances, strengthening the interpretation of tau-specific hippocampal pathology.

The present findings on trace eyeblink conditioning complement and extend our previous work examining spatial and object-recognition memory in rTg4510 mice [12]. In that study, we demonstrated that 6-month-old rTg4510 mice exhibit impairments in both Morris water maze performance and novel object recognition, and that these deficits are prevented by DOX-mediated suppression of tau expression initiated at 4 months of age. Critically, the same DOX treatment regimen that prevented spatial and object-recognition deficits also prevented trace eyeblink conditioning impairments in the present work, indicating a consistent therapeutic window for tau suppression across multiple memory domains. Furthermore, our previous Western blot analyses revealed that autophagy markers LC3A and LC3B were significantly elevated in 6-month-old rTg4510 mice alongside NFT accumulation [12], suggesting compensatory activation of protein clearance mechanisms during early pathological stages. The parallel emergence of deficits across spatial, non-spatial recognition, and temporal associative learning between 4 and 6 months of age suggests that this period represents a critical stage of tau-dependent hippocampal dysfunction, during which multiple memory systems become simultaneously vulnerable.

An important consideration when selecting behavioral paradigms for rTg4510 mice is the recent finding that visual cortical plasticity is disrupted in this model [33]. This observation suggests that non-spatial tasks, which rely less heavily on intact visual processing than spatial navigation paradigms such as the Morris water maze, may provide a more specific assessment of hippocampus-dependent cognitive function. Trace eyeblink conditioning, which depends primarily on auditory cues and somatosensory feedback rather than complex visual discrimination, is therefore particularly well-suited for isolating tau-related memory deficits from potential confounding effects of sensory impairment.

Importantly, the two non-spatial tasks differed in sensitivity. The neurobiological basis of eyeblink conditioning is well established, with the cerebellum playing a central role in delay eyeblink conditioning [34] and the hippocampus being essential for trace paradigms [35,36]. Trace eyeblink conditioning requires precise hippocampal–cerebellar coordination and dorsal CA3–CA1 circuitry to associate temporally separated stimuli, whereas contextual fear conditioning engages ventral hippocampal–amygdala networks that may temporarily buffer against early dysfunction [22,35,36,37]. This distinction explains why trace conditioning unmasked deficits earlier and more sensitively, and supports the interpretation that different hippocampal subcircuits are differentially vulnerable to tau pathology.

Interestingly, the relatively preserved performance in contextual fear conditioning during early phases suggests that certain functions may be transiently maintained through compensatory mechanisms. This interpretation aligns with established frameworks of cognitive reserve and scaffolding [38,39,40] and is further supported by functional magnetic resonance imaging evidence of compensatory hyperactivation in hippocampal and control networks [41,42].

At the circuit level, tau pathology is known to induce interneuron dysfunction and network hyperexcitability [43,44]. Recent studies have also demonstrated that neurodegenerative processes target large-scale brain networks in a systematic manner [44]. While these circuit-level changes were not directly measured in this study, such alterations provide a mechanistic substrate for transient compensation that ultimately fails, and for the high sensitivity of long-trace eyeblink conditioning, which critically depends on precise hippocampal–prefrontal timing. Our findings are consistent with earlier studies reporting that P301L tau transgenic mice develop NFTs, synaptic dysfunction, and memory impairments [9,45]. The rTg4510 model has been extensively validated, with the original studies demonstrating tau suppression effects on memory [10] and age-dependent pathological progression [11]. The P301L mutation, originally identified in frontotemporal dementia with parkinsonism-17 (FTDP-17), increases tau’s propensity for aggregation and reduces microtubule binding [46]. The fundamental mechanisms of tau pathology involve both its normal physiological functions and pathological aggregation processes [24,46]. In rTg4510 mice, this mutant tau undergoes progressive hyperphosphorylation and aggregation, leading to neuronal dysfunction and cell death [47]. The pathological cascade begins with disrupted microtubule dynamics and axonal transport, subsequently affecting synaptic integrity and ultimately culminating in cognitive decline [48]. The use of a long trace interval (500 ms) in the present study is particularly relevant, as longer stimulus-free intervals have been reported to be more sensitive in detecting hippocampus-dependent learning impairments in mice [20,21]. The experimental basis s for this sensitivity has been well established in the trace conditioning literature [29,30]. Thus, trace eyeblink conditioning may serve as a valuable complement to spatial tasks such as the water maze, revealing dysfunctions that emerge earlier or in different hippocampal subcircuits.

The temporal precision of learned responses, as reflected in response peak latency (Figure 2D), provides further insight into the neural mechanisms affected by tau pathology. Although we did not observe statistically significant differences in peak latency between groups, rTg4510 mice without DOX showed a trend toward shorter latency during early learning (Day 4), which normalized by Day 10. This pattern may reflect disrupted hippocampal-prefrontal timing coordination, as trace eyeblink conditioning critically depends on precise temporal encoding within hippocampal CA1-CA3 circuits [20,34,35] and their interaction with medial prefrontal cortex [49,50].

Notably, rTg4510 mice exhibit early electrophysiological alterations in frontal cortical layer 3 pyramidal neurons that precede morphological changes [51], as well as synaptic abnormalities in the prefrontal cortex, including reduced PSD-95 and altered EAAT2 expression [52]. These prefrontal deficits are distinct from typical amyloid-driven AD models and may specifically compromise the temporal processing required for trace conditioning. The subtle latency abnormalities observed during early learning, together with the significant acquisition deficit on Day 10, suggest that tau pathology progressively disrupts the hippocampal-prefrontal networks essential for temporally precise associative learning. This interpretation underscores the unique sensitivity of trace eyeblink conditioning to detect tau-related dysfunction in circuits that extend beyond the hippocampus proper.

The observed deficits further highlight the importance of tau pathology in disrupting cognitive functions beyond spatial memory. Tau accumulation accelerates synaptic dysfunction and neuronal loss [53,54], and has also been reported to correlate more closely with clinical decline than β-amyloid burden [6,55]. The propagation of pathological tau occurs through trans-synaptic mechanisms, with misfolded tau species being released from affected neurons and taken up by connected cells [56]. This prion-like spreading follows anatomical connectivity patterns, explaining the stereotypical progression of tauopathy across brain regions [57]. While tauopathies such as progressive supranuclear palsy (PSP) and corticobasal degeneration (CBD) occur independently of Aβ, AD combines both amyloid plaques and tau pathology [58]. This distinction underscores the caution required when extrapolating from tauopathy models to AD, but also strengthens the rationale for targeting tau directly in therapy.

While our study focused on behavioral readouts in a mouse model, the recent approval of lecanemab, an immunotherapy targeting Aβ protofibrils, has validated the principle of immunotherapy in AD [4,5]. This sets a precedent for tau-directed strategies, which could act by intercepting tau seeds during extracellular propagation. However, recent clinical trials of tau-directed immunotherapies such as gosuranemab and semorinemab have shown mixed results, highlighting the complexity of tau-mediated neurodegeneration [26,59,60,61,62]. Within this framework, behavioral readouts such as trace eyeblink conditioning may serve as translational biomarkers, enabling sensitive detection of early hippocampal dysfunction and evaluation of therapeutic efficacy. Critically, trace eyeblink conditioning represents a rare cross-species paradigm that employs identical experimental protocols in both rodents and humans, measuring comparable hippocampal-dependent learning processes—in marked contrast to conventional rodent assays such as water maze or contextual fear conditioning that lack direct human counterparts [63,64]—underscoring its potential to provide a practical paradigm for translational investigations of tau-related memory dysfunctions.

Several important limitations should be acknowledged. First, we did not perform sex-specific analyses, as both the present study and our previous work [12] employed mixed-sex groups (including both males and females) to maintain consistency with established behavioral protocols. Given emerging evidence for sex differences in tau pathology and behavioral outcomes in transgenic tauopathy models, future studies should systematically examine potential sex-dependent effects.

Second, detailed histopathological, biochemical, and molecular analyses were not included in the current study, as our primary aim was to establish a sensitive behavioral assay for tau-related dysfunction. Nevertheless, our previous work at the same time point demonstrated significant NFT accumulation and elevated autophagy markers, both correlated with behavioral deficits and prevented by DOX treatment [12], providing corroborating evidence for tau-dependent pathology. Future investigations combining trace eyeblink conditioning with concurrent molecular and electrophysiological assessments will be essential to elucidate the precise mechanisms underlying tau-related temporal processing deficits.

Third, while DOX treatment effectively suppresses tau expression, it may also influence gut microbiota composition [65,66], which could indirectly affect behavior. Several studies have shown that DOX administration can alter microbiome diversity in both transgenic and wild-type mice [66] and that these effects may interact with transgene expression to modulate behavioral outcomes [67]. However, the fact that WT mice showed no behavioral differences between DOX-treated and untreated groups in the present study argues against major confounding effects of DOX on the behavioral measures employed here.

Finally, the present study focused on behavioral outcomes as an integrated readout of tau-dependent hippocampal dysfunction. This approach reflects our view that behavior represents a complex emergent property of neural systems, and that reductionist attempts to directly correlate specific molecular or histological changes with behavioral phenotypes may oversimplify the underlying mechanisms. Nonetheless, the convergent findings across multiple behavioral domains—spatial memory [12], object recognition [12], and trace eyeblink conditioning (present study)—provide strong evidence that tau pathology produces widespread hippocampal network dysfunction during the critical 4–6 month developmental window in rTg4510 mice.

## 5. Conclusions

The present study demonstrates that rTg4510 mice exhibit deficits in hippocampus-dependent temporal associative learning alongside previously reported spatial and object-recognition impairments. Assessment of spontaneous home cage activity confirmed that these cognitive deficits occur without general motor impairment, supporting the specificity of tau-related hippocampal dysfunction. Trace eyeblink conditioning was particularly sensitive to detecting tau-related deficits, revealing impaired acquisition and subtle temporal processing abnormalities, whereas contextual fear conditioning showed milder impairments. Suppression of mutant human tau expression by DOX treatment initiated at four months of age prevented the emergence of these learning deficits, indicating that the behavioral abnormalities were associated with sustained tau expression. These findings support the use of trace eyeblink conditioning as a sensitive behavioral assay for tauopathy models and highlight its potential as a translational biomarker. As a rare paradigm that employs identical protocols in rodents and humans, trace eyeblink conditioning offers promise for the early detection of hippocampal dysfunction and the evaluation of tau-targeted therapeutic interventions.

## Data Availability

The data are available upon request from the corresponding authors. These data are not publicly available due to institutional restrictions.

## Abbreviations

The following abbreviations are used in this manuscript:

Aβ	β-amyloid
AD	Alzheimer’s disease
APP	amyloid-β precursor protein;
CR	conditioned response;
TI	trace interval
